# Potentiodynamic Polarization Studies and Surface Chemical Composition of Bismuth Titanate (Bi*_x_*Ti*_y_*O*_z_*) Films Produced through Radiofrequency Magnetron Sputtering

**DOI:** 10.3390/ma6104441

**Published:** 2013-10-08

**Authors:** José E. Alfonso, Jhon J. Olaya, Manuel J. Pinzón, José F. Marco

**Affiliations:** 1 Grupo de ciencia de materiales y superficies, Universidad Nacional de Colombia, Bogotá AA 14490, Colombia; E-Mails: jjolayaf@unal.edu.co (J.J.O.); mjpinzonc@unal.edu.co (M.J.P.); 2 Associate Researcher of Centro Internacional de Física (CIF), Bogotá 14490, Colombia; 3 Departamento de Ingeniería Mecánica y Mecatrónica, Universidad Nacional de Colombia, Bogotá AA 14490, Colombia; 4 Instituto de Química-Física “Rocasolano”, CSIC, c/ Serrano 119, Madrid 28006, Spain; E-Mail: jfmarco@iqfr.csic.es

**Keywords:** RF Magnetron Sputtering, thin films, corrosion resistance, morphology

## Abstract

The applications of Bismuth Titanate (Bi*_x_*Ti*_y_*O*_z_*) materials have been focused on their electronic and optical properties, but with respect to the use of these compounds in applications like corrosion resistance, have been very few or nonexistent. For this reason, in the present investigation Bi*_x_*Ti*_y_*O*_z_* thin films were deposited using RF magnetron sputtering onto silicon wafers, stainless steel 316L, and titanium alloy (Ti_6_Al_4_V) substrates, in order to carry out a study of the corrosion behavior of this compound. The structural properties of the coatings were studied through X-ray diffraction (XRD), the morphology was determined using Scanning Electron Microscopy (SEM), the corrosion resistance behavior of the coated and uncoated substrates was evaluated via the Potentiodynamic Polarization technique, and surface chemical composition was evaluated through X-ray photoelectron spectroscopy (XPS). The XRD results indicated that the films were amorphous. The SEM micrographs showed that the deposited films were homogeneous, but in some cases there were cracks. The potentiodynamic polarization technique showed that the corrosion current in the coated substrates decreased by an order of two magnitudes with respect to the uncoated substrates, but in both cases the corrosion mechanism was pitting due to the pores in the film. The XPS analysis shows that the deposited films contain both Bi^3+^ and Ti^4+^.

## 1. Introduction

Bismuth Titanate (Bi*_x_*Ti*_y_*O*_z_*) compounds have been the subject of many studies due to their ferroelectric, piezoelectric and optical properties, which make them interesting materials for the fabrication of optical devices, ferroelectric memories, and lead-free piezoelectric sensors and actuators [1,2]. For instance, Bismuth Titanate (Bi_4_Ti_3_O_12_) is a material with an Aurivillius crystal structure, which in turn is composed of fluorite-type layers and perovskite-type layers. At approximately 948 K, the compound undergoes a phase transition, from a ferroelectric to a paraelectric phase. Within the Aurivillius family, this compound is the object of a lot of attention, due to its interesting ferroelectric properties, which make it a formidable candidate for the nonvolatile memory and dynamic memory of computers [3]. The production of this configuration of Bismuth Titanate covers a large number of techniques both physical vapor deposition (PVD) and chemical vapor deposition (CVD) and forms (from thin films to ceramic forms). This material exhibits an anisotropic behavior, which opens even more the possibilities for fabricating new forms of it, for instance Bi_4_Ti_3_O_12_ ceramics with grains aligned in certain directions through the tape-casting technique or those created by employing external parameters such as magnetic fields or pressure [4,5,6]. Another configuration of Bismuth Titanate compounds is the called the Pyrochlore structure (Bi_2_Ti_2_O_7_), which don't exist in the Bi_2_O_3_/TiO_2_ equilibrium phase diagram [7]. Several morphologies of the Bismuth Titanate Pyrochlore structure, such as nanowires, nanospheres, nanoparticles, nanotubes, *etc.*, have been reported by many authors, and also a large number of fabrication techniques have been documented, such as the co-precipitation method and the template-free hydrothermal process, inter alia [8]. Research on applications for this phase of Bismuth Titanate is focused on photocatalysis, due to the fact that it is capable of decomposing a wide variety of organic and inorganic pollution and toxic materials, which might solve both environmental and energy problems in the future [9]. However, exploitation of the rest of the properties of this compound has been object of research for some groups; for instance, its high permittivity and significantly low current leakage make it a promising alternative for gate insulating layers in advanced MOS transistors [10]. Besides the two Bismuth Titanate phases described above, another phase of this material exists, the Bi_20_TiO_12_ phase. This phase belongs to the sillenite family compounds. This phase, together with the above-named Bi_4_Ti_3_O_12_ and Bi_2_Ti_4_O_11_, are the only equilibrium phases among the large variety of Bismuth Titanate compounds; however, the Bi_12_TiO_20_ phase, like the Bi_2_Ti_2_O_7_ phase, has interesting photocatalytic properties, this last being, as said before, a phase that is nonexistent in the Bi_2_O_3_/TiO_2_ equilibrium phase diagram [11,12]. For this compound, besides its photocatalytic properties, possible applications related to its electro-optic, acousto-optic, and piezoelectric properties have been researched, and it is also important to note that this phase exhibits a higher sensitivity for red light and a lower optical activity Q, which is favorable for the development of many devices [13].

Although the structural behavior, optical properties, photoconductivity, and dielectrical properties have been extensively studied, the number of studies devoted to the corrosion resistance of these materials is still small. In the present paper, we present preliminary results with respect to the corrosion resistance of amorphous bismuth Titanate.

## 2. Experimental Procedure

**Figure 1:**
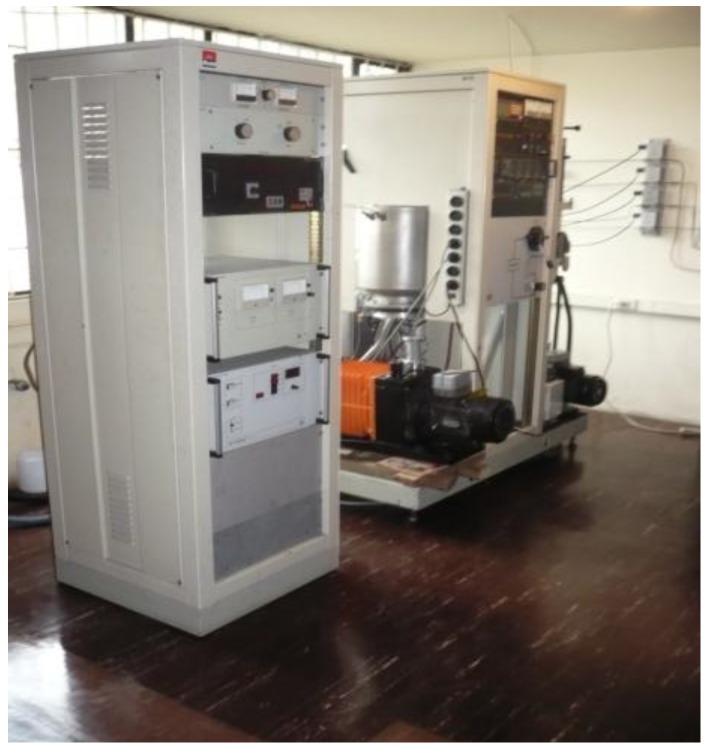
Alcatel HS 2000 RF sputtering system.

**Figure 2:**
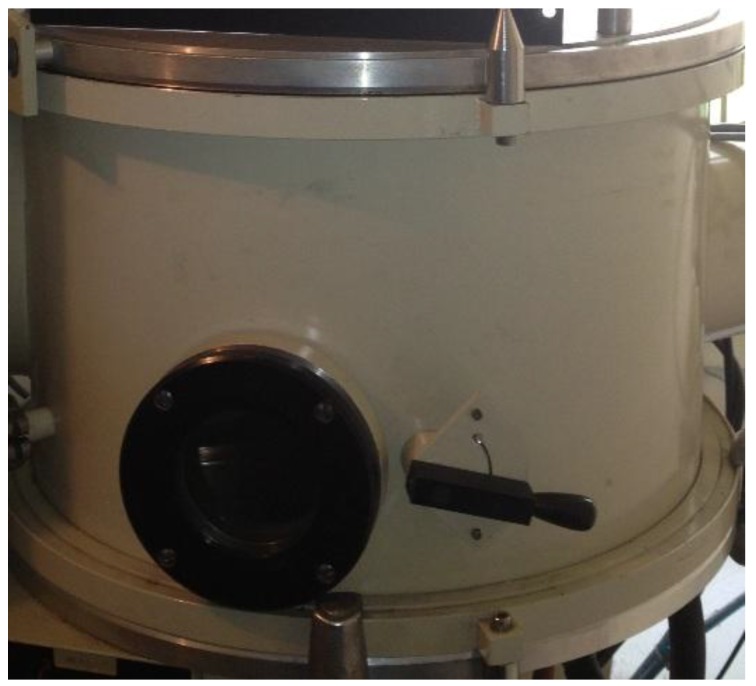
Deposition chamber.

The equipment used to grow Bi*_x_*Ti*_y_*O*_z_* thin films was an Alcatel HS 2000 RF sputtering system with a balanced magnetron 101.6 mm in diameter (see Figure 1 and Figure 2), which was described in a previous paper [14]. The Bi*_x_*Ti*_y_*O*_z_* thin films were obtained from a 101.6 mm × 6.35 mm Bi_4_Ti_3_O_12_ (99.9%) target (Plasma Materials). The parameters used during the deposition process were: base pressure 4.0 × 10^−3^ Pa, total working pressure 7.4 × 10^−1^ Pa, deposition time half an hour, and target-substrate distance 50.8 mm. We studied the influence of several deposition parameters, such as power supplied to the target (from 50 W to 200 W), substrate temperature (which varied from 293 K to 623 K), and argon (99.999%) flow (from 5 sccm to 25 sccm). The structural characterization of the thin films was performed through X-ray diffraction (XRD) with a Phillips diffractometer operated at 30 kV and 20 mA, working in the Bragg-Brentano configuration and using Cu Kα radiation. X-ray photoelectron spectroscopy (XPS) data were recorded using a CLAM2 analyzer under a vacuum better than 1 × 10^−6^ Pa using Mg Kα radiation and constant pass energy of 20 eV. The binding energy scale was calibrated using the C 1s signal from the adventitious carbon layer, which was set at 284.6 eV. The equipment used to carry out the potentiodynamic polarization tests was a Gamry Instruments reference 600 potentiostat, which serves also as a galvanostat, zero resistance ammeter, and frequency analyzer. The reference electrode used in this test was a Saturated Calomel Electrode (SCE), accompanied of a Platinum electrode; and as a work electrodes were used the samples. The preparation of the samples was carried out in the following order: cleaning of samples by immersing them in isopropyl alcohol in an ultrasonic bath for 5 min, drying (with dry air), mounting of samples on the galvanic cell, and finally placing the galvanic cell inside a Faraday cage in order to minimize the effects of the magnetic and electrical fields in the environment. Analysis of the potentiodynamic polarization curves was done with Gamry Echem Analyst software.

## 3. Results and Discussion

XRD patterns recorded from the films grown onto silicon wafers, stainless steel 316L, and titanium alloy (Ti_6_Al_4_V) substrates exhibited the amorphous phase, this is probably due to the thermodynamic conditions, which did not allow the formation of a crystalline phase.

**Figure 3:**
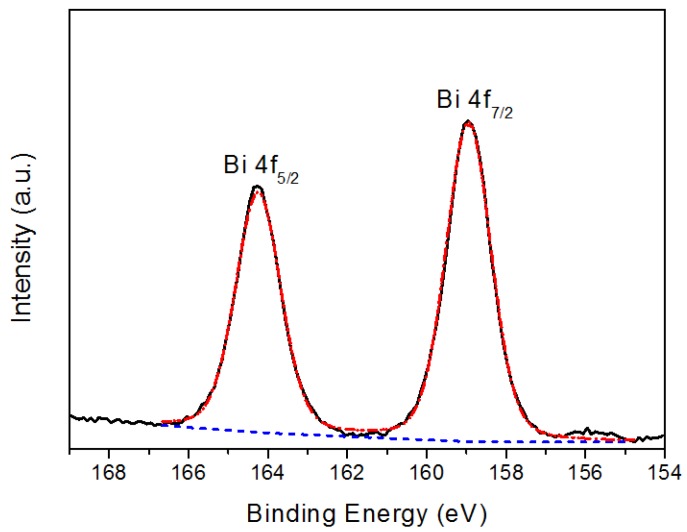
Bi 4f X-ray photoelectron spectroscopy (XPS) spectrum recorded from the coating.

**Figure 4:**
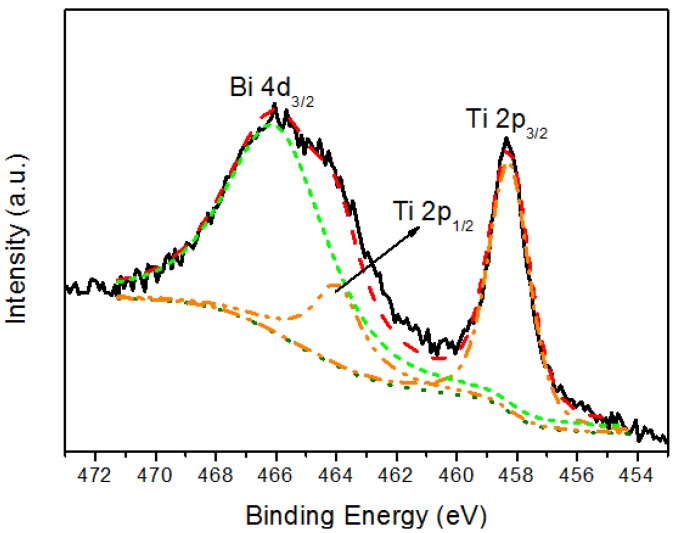
Ti 2p XPS spectrum recorded from the coating.

**Figure 5:**
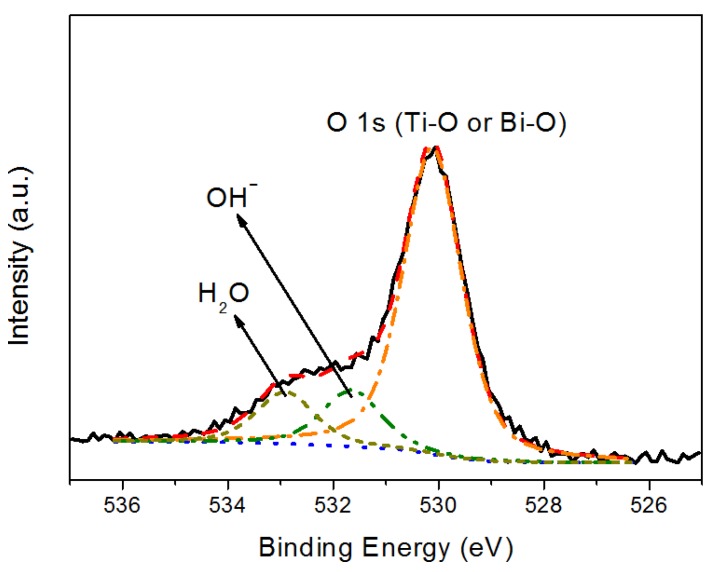
O 1s XPS spectrum recorded from the coating.

Figure 3, Figure 4 and Figure 5 show representative XPS spectra recorded from the Bi*_x_*Ti*_y_*O*_z_* films grown. Figure 3 shows a Bi 4f high resolution spectrum; this spectrum consists of a spin-orbit doublet with binding energies of 158.9 eV (Bi 4f_7/2_) and 164.2 eV (Bi 4f_5/2_). These binding energies are very similar to those characteristic of Bi_2_O_3_ [15], and are compatible with the presence of Bi^3+^ in the coating. Figure 4 shows a Ti high resolution spectrum; this spectrum consists of a spin-orbit doublet with binding energies of 458.3 eV (Ti 2p_3/2_) and 464.0eV (Ti 2p_1/2_). These binding energies are typical of TiO_2_ [16], and therefore the result indicates that the coating contains Ti^4+^. Additionally, this spectrum shows another peak centered at 465.0 eV that corresponds to the Bi 4d_3/2_ core level [17,18] of Bi that overlaps with the Ti 2p spectrum. Finally, the XPS O 1s data are shown in Figure 5. The spectrum was fitted considering three contributions: a dominant one located at 530.1 eV, characteristic of metal-oxygen bonds (Ti–O, Bi–O) [15,19], a second one located at 531.7 eV, which is typical of OH– groups [20], and a third at 532.9 eV, which is compatible with the presence of water absorbed into the film [21]. Therefore, although the XRD data do not show peaks that could be attributed to any mixed bismuth-titanium oxide, the XPS data demonstrate that the coatings contain both Bi^3+^ and Ti^4+^ and are therefore compatible with the presence of such an oxide. Unfortunately, the strong overlap of the Bi 4d_3/2_ peak with the Ti 2p spectrum precluded accurate determination of the stoichiometry of the deposited phase. These results were obtained for the coating deposited on the titanium alloy, stainless steel 316L, and silicon wafers.

We would like to remark that the XPS data recorded from the materials subjected to the corrosion tests are essentially the same as those recorded from the freshly-coated materials, indicating that the deposited oxides are chemically inert and do not suffer degradation under the corrosion conditions used.

**Figure 6:**
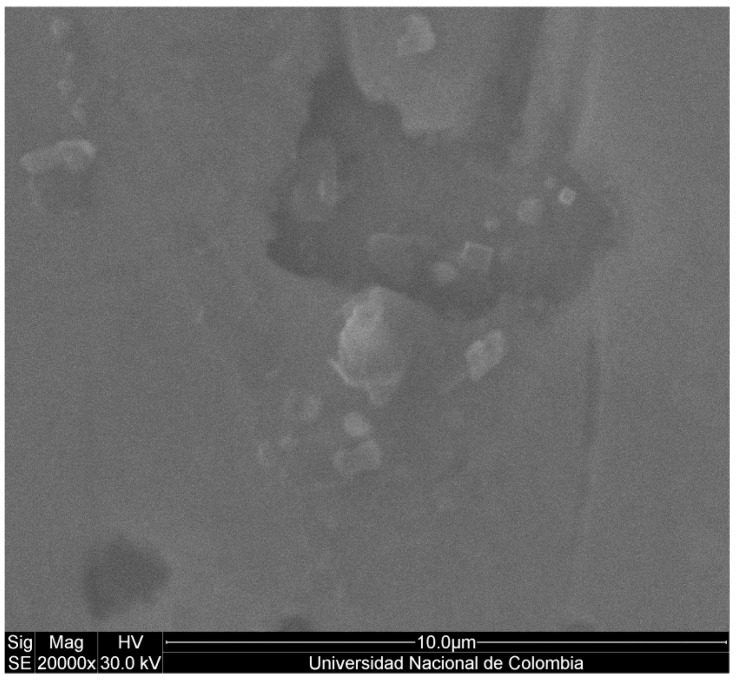
Scanning Electron Microscopy (SEM) micrograph of the Surface films.

Figure 6 shows the surface of the films. The micrograph allows us to determine that the surface of the films is formed by pores of 5 μm of average size cracks and not congruently melted material.

**Figure 7:**
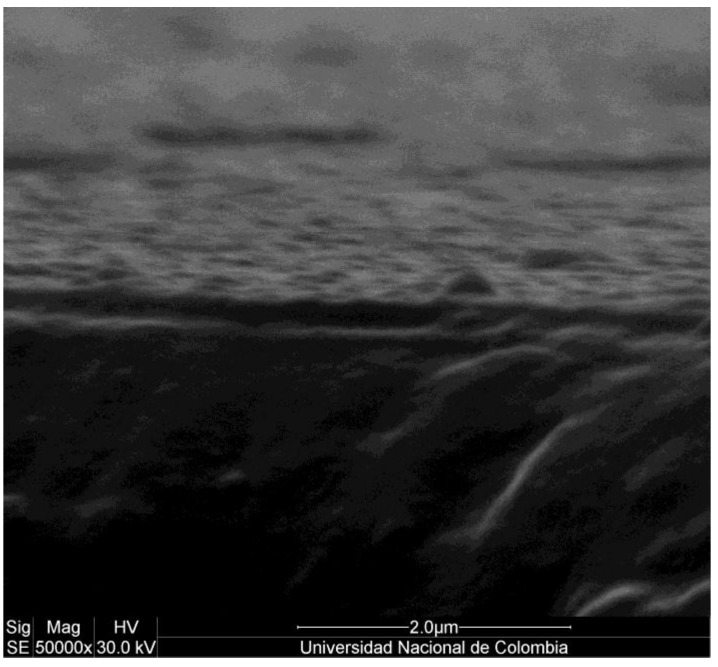
SEM micrograph of the transverse section of films.

Figure 7 shows the transverse section of the films. The micrograph allows us to establish that the films have an average thickness of 233 nm.

**Figure 8:**
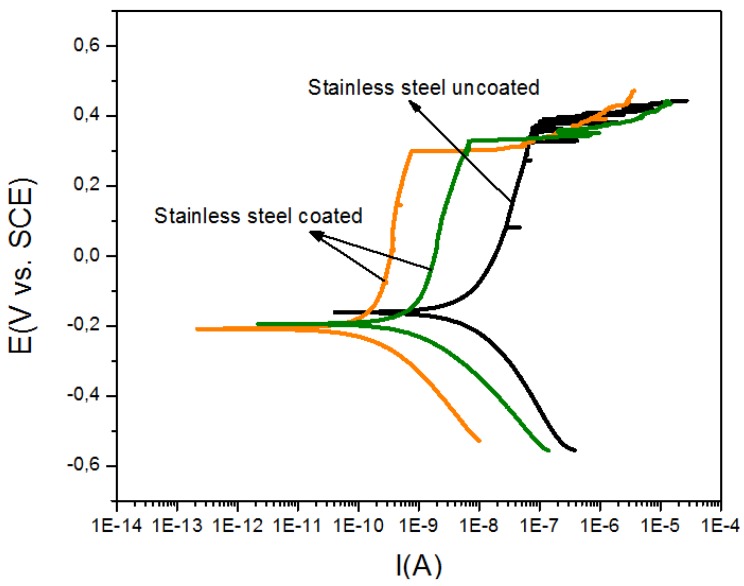
Potentiodynamic polarization curves recorded from stainless steel 316L coated (green and orange lines) and uncoated (Black line).

**Figure 9:**
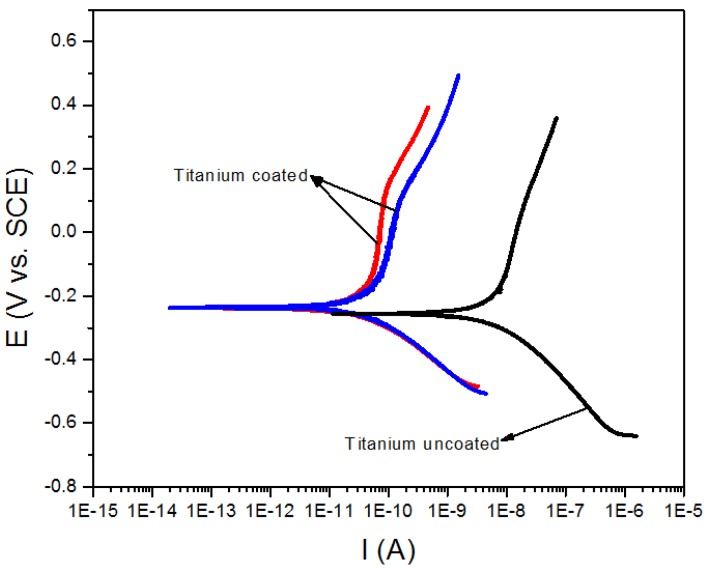
Potentiodynamic polarization curves recorded from the titanium alloy (Ti_6_Al_4_V) coated (Blue and red lines) and uncoated (Black line).

Figure 8 and Figure 9 show the anodic and cathodic polarization curves recorded from the coated and uncoated substrates, respectively, in NaCl (3%) solution for a period of 30 min. The potentiodynamic polarization measurements taken at 2 mV/s are shown in Table 1.

**Table 1:** Values of the potentiodynamic polarization measurements.

Sample	I_corr_ (nA)	E_corr_ (mV)	Corrosion rate (mm/y)
Stainless Steel 316L uncoated	10.60	−161	6.299 × 10^−4^
Stainless Steel 316L coated	1.840	−193	1.097 × 10^−4^
Titanium Alloy uncoated	6.960	−247	4.166 × 10^−4^
Titanium Alloy coated	0.028	−226	1.676 × 10^−6^

These results indicate that the Bi*_x_*Ti*_y_*O*_z_* coating that was produced exhibits a significant decrease in corrosion current as compared with that observed in the bare substrates. This decrease is of about one order of magnitude in stainless steel and two orders of magnitude in the titanium alloy.

Moreover, the corrosion rate was estimated to decrease about two orders of magnitude in stainless steel and approximately three orders of magnitude in the titanium alloy. These values suggest that Bi*_x_*Ti*_y_*O*_z_* films have a considerable corrosion protection effect. However, the corrosion mechanism is due to the crevice corrosion effect, which is a consequence of the presence of cracks in the surface of the films as evidenced in SEM micrographs. These cracks allow the electrolyte to penetrate to the substrate and attack it.

## 4. Conclusions 

Bi*_x_*Ti*_y_*O*_z_* amorphous thin films were grown, and their corrosion resistance was assessed. The preliminary results allowed us to establish that this coating is potentially useful as a protective layer for materials or tools that function in chemically aggressive environments.
